# Clinical Characterization and Outcomes of Pneumonic Plague in Madagascar: A Case Series

**DOI:** 10.1093/ofid/ofag635

**Published:** 2026-10-08

**Authors:** Mihaja Raberahona, Lisy Hanitra Razananaivo, Tansy Edwards, Tsinjo Fehizoro Razafindrantsinana, Reziky Tiandraza Mangahasimbola, Theodora Mayouya-Gamana, Ravaka Randriamparany, Voahangy Andrianaivoarimanana, Minoarisoa Rajerison, Josephine Bourner, Rindra Randremanana, Peter Horby, Piero Olliaro

**Affiliations:** Équipe de Recherche Clinique en Maladies Infectieuses, University Hospital Joseph Raseta Befelatanana, Antananarivo, Madagascar; Centre d’Infectiologie Charles Mérieux, University of Antananarivo, Antananarivo, Madagascar; Institut Pasteur de Madagascar, Antananarivo, Madagascar; Global Health Trials Unit, Department of Clinical Sciences, Liverpool School of Tropical Medicine, Liverpool, UK; Department of Infectious Disease Epidemiology, London School of Hygiene and Tropical Medicine, London, UK; Institut Pasteur de Madagascar, Antananarivo, Madagascar; Institut Pasteur de Madagascar, Antananarivo, Madagascar; Institut Pasteur de Madagascar, Antananarivo, Madagascar; Institut Pasteur de Madagascar, Antananarivo, Madagascar; Institut Pasteur de Madagascar, Antananarivo, Madagascar; The Biodiscovery Institute, School of Life Sciences, University of Nottingham, Nottingham, UK; Centre d’Infectiologie Charles Mérieux, University of Antananarivo, Antananarivo, Madagascar; ISARIC, Pandemic Sciences Institute, Nuffield Department of Medicine, University of Oxford, Oxford, UK; Institut Pasteur de Madagascar, Antananarivo, Madagascar; ISARIC, Pandemic Sciences Institute, Nuffield Department of Medicine, University of Oxford, Oxford, UK; ISARIC, Pandemic Sciences Institute, Nuffield Department of Medicine, University of Oxford, Oxford, UK

**Keywords:** Madagascar, plague, pneumonic plague

## Abstract

Pneumonic plague is rare compared to bubonic plague; data on clinical presentation and treatment outcomes are scant. We report on 13 prospectively-enrolled suspected pneumonic plague cases (7 laboratory-confirmed, one probable, 5 not-plague) treated with aminoglycoside plus ciprofloxacin for 10 days and discuss findings in the context of the available literature.

Plague is caused by the gram-negative bacterium *Yersinia pestis*. Clinical manifestations of plague are bubonic (acquired through a flea bite), septicemic (complication of uncontrolled bubonic plague), and pneumonic (occurring either as progression of bubonic plague or as primary infection via airborne transmission from an infected individual) [1].

Pneumonic plague is rare compared to bubonic plague, with extremely limited data relating to clinical characteristics and patient outcomes. A recent systematic review identified 29 articles between 1945 and 2017 reporting treatment outcomes for only 108 cases of confirmed or probable pneumonic plague [2]. Plague is endemic in Madagascar—the country that reports the most plague cases worldwide [3]—with seasonal outbreaks in remote rural settings of essentially bubonic plague, except an unusual urban outbreak of pneumonic and bubonic plague in 2017, with 1878 notified pneumonic plague cases, of which 386 (21%) were identified as probable and 32 (2%) were confirmed [4].

## MATERIALS AND METHODS

During the IMASOY randomized controlled trial for treatment of plague (NCT04110340) [5], suspected pneumonic plague cases of all ages were enrolled separately to suspected bubonic cases, under the following criteria: recent onset (<7 days) of fever or history of fever, cough, and tachypnea (respiratory rate [RR] >24 breaths/minute in adults and age-specific in children), and an epidemiological link with primary or secondary pneumonic plague within 7 days of onset of symptoms (Supplementary Information 1). The treating physician decided on clinical grounds whether pneumonic plague was suspected and initiated treatment accordingly. Concomitantly, they sent a sputum sample to the Central Plague Laboratory, hosted by Institut Pasteur de Madagascar (IPM) for confirmation, which included (1) an anti-F1 lateral flow test; (2) a 2-step molecular diagnosis using first real-time polymerase chain reaction (PCR) targeting 2 fragments of the *pla* and *caf-1* genes of *Y pestis*, followed, for samples with inconclusive results, by conventional PCR targeting 3 genes (*pla*, *caf-1*, and *inv1100*); (3) bacteriological diagnosis with direct culture and amplification in mice, followed by biochemical identification of the suspected strain by API 20E and by bacteriophage lysis test; and (4) serological diagnosis by enzyme-linked immunosorbent assay (anti-F1 antibodies, immunoglobulin G) on blood samples taken on days 1, 11, and 21. Confirmation of *Y pestis* infection and plague case classification were based on the World Health Organization criteria [6]. Details of the protocol including clinical and laboratory procedures, definitions, and endpoints can be found in Randremanana et al [7].

Initially, treatment was via random allocation to a 10-day regimen of aminoglycoside plus ciprofloxacin (streptomycin 1 g intramuscularly twice daily [BID] in adults, 15 mg/kg BID in children on days 1–5, or gentamicin 2.5 mg/kg intravenously on days 1–5, alongside ciprofloxacin on days 1–10), or monotherapy with ciprofloxacin (500 mg orally BID in adults, 15 mg/kg BID in children on days 1–10) (Supplementary Information 2). Following a change in national treatment recommendations for pneumonic plague, all subsequent cases were offered gentamicin (days 1–5) plus ciprofloxacin (days 1–10), in a change to observational cohort follow-up [8]. Treatment response was assessed at day 11 (end-of-treatment assessment) and on day 21 for safety and clinical status. As per the protocol, “unfavorable outcome” was defined as death or persisting fever or tachypnea at day 11.

## RESULTS

Out of 137 cases initially screened, 102 had a clinical diagnosis other than pneumonic plague and 22 could not be followed up—in 19 because of critical condition and in 3 because enrollment was suspended due to extreme weather conditions. Of the remaining 13, 7 (61.5%) were laboratory-confirmed (2 positive on PCR and culture, 5 PCR positive only), 1 was probable, and 5 were negative for *Y pestis* by either PCR or culture (Supplementary Figure 1).

Details on demographics, presenting symptoms, treatment, efficacy, and tolerability are presented in Table 1.

**Table 1.** Participant Data

ID	Diagnosis	Sex, Age	Presenting Symptoms^a^ (No. of Days)	RR	HR	SBP	DBP	SpO_2_	Initial Treatment	Additional Treatment	Outcome D11^b^	MedDRA Preferred Term	Type	Onset Day	Relation	Intensity
1	Probable	M 32	Fever (3) Cough (3) Dyspnea (0) Hemoptysis (0) Chest pain (0) Headache	36	126	100	60	91%	G + C	…	Unfavorable: Tachypnea D11, resolved D21	…	…	…	…	…
2	Confirmed^c^	F 30	Fever (2) Cough (2) Dyspnea (1) Chest pain (2) Headache	38	62	100	60	75%	Randomized to C	…	Unfavorable: Tachypnea D11, resolved D21	…	…	…	…	…
3	Confirmed^d^	M 6	Fever (3) Cough (1) Chest pain (2) Headache	30	89	100	60	NA	Randomized to C	G: D2, D4–D9	Favorable	Dyspnea	SAE	2	None	Moderate
												Chest pain	SAE	2	Possible	Moderate
												Vomiting	AE	3	Possible	Mild
												Pyrexia	AE	3	None	Moderate
4	Confirmed^d^	M 49	Fever (2) Cough (2) Dyspnea (1) Chest pain (1) Headache	30	89	96	53	99%	Randomized to G + C	G: D6	Unfavorable: Tachypnea D11, resolved D21	…	…	…	…	…
5	Confirmed^d^	F 74	Fever (1) Cough (1) Dyspnea (1) Chest pain (1) Headache	30	88	110	73	NA	Randomized to G + C	G: D6	Favorable	Hypertension	AE	10	None	Mild
6	Confirmed^d^	M 32	Fever (1) Cough (1) Dyspnea (1) Hemoptysis (1) Chest pain (1) Headache	31	115	100	60	NA	Randomized to C	G: D1–D5	Favorable	Altered state of consciousness, pyrexia	AE	4	None	Mild
													AE	5	None	Mild
7	Confirmed	F 56	Fever (2) Cough (2) Dyspnea (2) Hemoptysis (1) Headache	50	107	90	60	88%	G + C	…	Unfavorable: Tachypnea D11, resolved D21	…	…	…	…	…
8	Confirmed	F 51	Fever (2) Cough (1) Dyspnea (0) Hemoptysis (0) Chest pain (0) Headache	26	126	80	60	79%	G + C	…	Unfavorable: Died D2	ARDS	SAE	1	None	Fatal
9	Suspected (not plague)	M 10	Fever (2) Cough (2) Dyspnea 2) Chest pain (2)	33	104	120	80	94%	Randomized to S + C	…	Favorable	…	…	…	…	…
10	Suspected (not plague)	M 40	Fever (1) Cough (1) Dyspnea (1) Hemoptysis (1) Chest pain (1) Headache	30	120	100	70	92%	Randomized to G + C	…	Unfavorable: Tachypnea D11, resolved D21	…	…	…	…	…
11	Suspected (not plague)	M 22	Fever (4) Cough (4) Dyspnea (4) Hemoptysis (4) Chest pain (4) Headache	30	80	100	60	NA	G + C	…	Favorable	…	…	…	…	…
12	Suspected (not plague)	M 35	Fever (6) Cough (3) Dyspnea (1) Hemoptysis (3) Chest pain (3) Headache	32	80	120	80	90%	G + C	…	Favorable	…	…	…	…	…
13	Suspected (not plague)	M 19	Fever (2) Cough (2) Dyspnea (2) Hemoptysis (1) Chest pain (2) Headache	42	89	100	60	88%	G + C	…	Unfavorable: Tachypnea D11, resolved D21	…	…	…	…	…

Patients randomized to treatment, prior to change to observational cohort: 2, 3, 4, 5, 6, 9, and 10.

Treatment: C = ciprofloxacin for 10 days (D1–10); G + C = gentamicin D1–5, ciprofloxacin D1–10; S + C = streptomycin D1–5, ciprofloxacin D1–10. Extra treatment on days indicated: G, gentamycin; C, ciprofloxacin; S, streptomycin.

Safety: Where AEs were experienced by participants, all AEs are shown in the final columns.

Abbreviations: AE, adverse event; ARDS, acute respiratory distress syndrome; D, day; DBP, diastolic blood pressure (mm Hg); F, female; HR, heart rate (beats/min); M, male; MedDRA, Medical Dictionary for Regulatory Activities; NA, not available; RR, respiratory rate (breaths/min); SAE, serious adverse event; SBP, systolic blood pressure (mm Hg); SpO_2_, peripheral oxygen saturation.

^a^Duration of symptoms in days preceding day enrolled, where symptom not shown means not reported by the participant.

^b^For unfavorable outcome, all observed reasons given.

^c^Presented also with enlarged lymph node(s).

^d^Cluster in the same hamlet.

The median age was 32 years overall and 40.5 years among confirmed cases (range, 6–74 years). The female-to-male ratio was 4:9 and 4:4, respectively.

Presenting symptoms were similar between confirmed, probable, and suspected (not confirmed) cases. All 13 patients had recent onset of symptoms and presented with fever lasting between 1 and 6 days and cough. All 8 confirmed/probable cases also had headache, 7 had dyspnea and chest pain, and 4 hemoptysis. On the AVPU (alert, verbal, pain, unresponsive) scale, 6 were alert and 2 capable of verbal response. Two had taken ampicillin prior to being diagnosed and treated for plague. The median RR, heart rate (HR), systolic blood pressure (SBP), diastolic blood pressure (DBP), and peripheral capillary oxygen saturation (SpO_2_) were 30.5 breaths/minute, 98 beats/minute, 100 mm Hg, 60 mm Hg, and 88%, respectively, among confirmed/probable cases. The respective values for suspected cases were 32 breaths/minute, 89 beats/minute, 100 mm Hg, 70 mm Hg, and 91%, respectively. There was a cluster of 4 confirmed pneumonic plague cases from the same hamlet. Two cases presented also with enlarged lymph node(s), 1 suspected, 1 confirmed—the latter part of a cluster with another 2 bubonic plague cases.

By day 11, 1 of the 7 confirmed patients had died, and 4 additional patients (3 confirmed, 1 probable) remained symptomatic with tachypnea but no other symptoms; all of them were asymptomatic on day 21, without additional treatment. Of the 5 noncases, none died and 2 were still tachypneic on day 11 but asymptomatic on day 21. The end-of-treatment day 11 unfavorable outcome among confirmed/probable cases was 5 of 8 (63% [95% confidence interval {CI}, 29%–96%]).

The fatal outcome occurred on day 2 from respiratory failure in a female participant aged 51 years who presented with tachypnea (RR, 26 breaths/min), tachycardia (HR, 126 beats/min), indication of shock (SBP, 80 mm Hg; DBP, 60 mm Hg; mean arterial pressure, 67 mm Hg, shock index [HR/SBP] = 1.6), and hypoxemia (SpO_2_ = 79%).

Three patients reportedly suffered from 1 serious adverse event (SAE) each: 1 was the above-mentioned death (unrelated to treatment), 1 mild chest pain (possibly related), and 1 moderate dyspnea (unrelated). This latter patient also had 2 treatment-emergent non-SAEs (TEAEs; vomiting and pyrexia). An additional 3 patients had 1 TEAE each (pyrexia, altered state of consciousness, and hypertension).

## DISCUSSION

Individual patient data relating to presentation and outcomes in suspected, probable, or confirmed pneumonic plague are extremely rare and not well documented [2]. Given the small numbers reported in the literature, and in the absence of evidence from randomized controlled trials, guidance on pneumonic plague treatment is based on information from case series. Though it is not possible to draw definite conclusions from this small study, these data offer some potentially interesting indications, in conjunction with prior data.

Untreated, pneumonic plague is a highly fatal disease: In preantibiotic times in India, 71 deaths were documented among 83 pneumonic cases (case-fatality rate [CFR], 86% [95% CI, 78%–93%]) [9], and a recent systematic review covering the period 1945–2017 [2] reports a pooled CFR of 97% (95% CI, 92%–99%) in untreated pneumonic plague, compared to a pooled CFR of 16% (95% CI, 10%–23%) in those who received antibiotic treatment. During the 2017 urban outbreak in Madagascar, the CFR among confirmed pneumonic plague cases was 25% (95% CI, 13%–42%) (8/32), compared to 7.2% (95% CI, 5%–10%) among probable pneumonic cases (27/360), and similar to the 24% (95% CI, 15%–36%) mortality observed in confirmed bubonic plague (16/66) [4]. During 2018–2025, 195 cases of suspected pneumonic plague were notified in Madagascar, of which 13 (7%) were enrolled in the IMASOY trial (2020–2024) and 182 (93%) were treated within normal routine, of which, respectively, 7 (54%) and 73 (40%) were confirmed and 1 and 11 probable plague. The CFR among the confirmed pneumonic cases treated in routine settings outside of the trial was 19% (95% CI, 12%–30%) (14/73) (information summarized in Figure 1). A note of caution with these data is that the definition of “pneumonic” plague varies with the diagnostics available; therefore, these should not be interpreted as directly comparable estimates.

**Figure 1.**
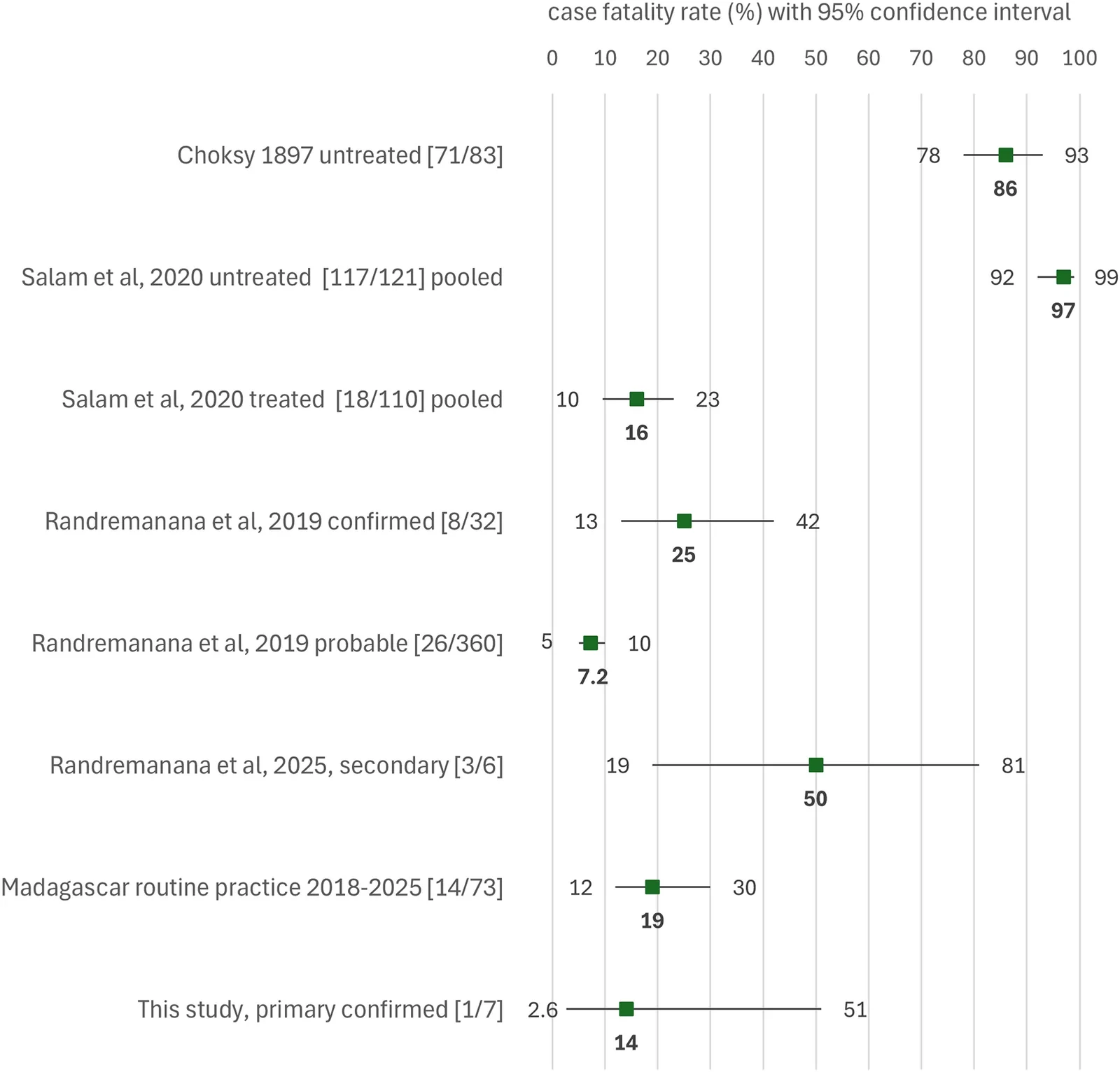
Reported case fatality rates of pneumonic plague. Case fatality rates are reported as percentage with Wilson score 95% confidence interval. Data are extracted from the respective papers. Numbers in square brackets denote number of deaths over number of cases reported.

With the proviso that these are small numbers, laboratory-confirmed primary pneumonic plague in this study resulted in a 14% CFR (with a wide 95% CI ranging from 2.6% to 51% given the small sample size). The exclusion of critically ill cases with pneumonia who could not undertake diagnostic procedures for plague may have biased mortality and clinical outcomes estimation. None of the suspected, but not confirmed, cases of plague died—possibly on the account of the broad-spectrum activity of aminoglycosides and fluoroquinolones thus likely to cover common bacterial causes of lower respiratory tract infections.

There is no specific information from the literature or routine data about the proportion of cases presenting as bubonic plague that evolve to pneumonic plague and the associated mortality. In the IMASOY trial, 6 of the 222 laboratory-confirmed bubonic plague cases (2.7%) developed pneumonic plague, and 3 of them died (CFR, 50% [95% CI, 19%–81%]) (Figure 1) with signs of bloodstream dissemination and septicemic shock.

As also shown by this study, presenting conditions of pneumonic plague appear to overlap with those of typical lower respiratory tract infections, making differential diagnosis on clinical grounds challenging. In the period 2018–2025, the Central Plague Laboratory received 117 sputum samples of clinically suspected pneumonic plague, 44 of which (38%) were confirmed by PCR but only 7 of the 44 were also positive on culture (Cohen's κ = 0.19). A point-of-care test that could reliably rule in and rule out pneumonic plague is not available. The anti-F1 antigen–based lateral flow test produced by IPM on those 117 samples showed a sensitivity of 98% and specificity of 89%, which translates in a false-detection rate of 16% and a false-omission rate of 2% at the observed 38% incidence.

Given its severity, pneumonic plague must be rapidly identified and treated. Equally critical is to detect early signs of respiratory compromise in patients with bubonic plague and adapt treatment accordingly.

## Supplementary Material

ofag635_Supplementary_Data

## Supplementary Data

Supplementary materials are available at *Open Forum Infectious Diseases* online. Consisting of data provided by the authors to benefit the reader, the posted materials are not copyedited and are the sole responsibility of the authors, so questions or comments should be addressed to the corresponding author.
