# Supplementary material for: Clinical Characterization and Outcomes of Pneumonic Plague in Madagascar: A Case Series
Source: Open Forum Infect Dis. 2026 Oct 8;13(10):ofag635. doi: 10.1093/ofid/ofag635 (PMC13646609; doi:10.1093/ofid/ofag635)
Supplement: Supplementary file 1 [file ofag635_supplementary_data.zip › OFID-D-26-00663_R1_Supplementary Information.docx]

**Supplementary Information**

1. **Case Definitions: Pneumonic plague**

| **Confirmed case** | **Probable case** | **Suspected case** |
| --- | --- | --- |
| Using sputum samples   - qPCR is positive on D1 sample or - culture is positive on D1 sample | Using sputum samples   - RDT (conducted at central laboratory) positive on D1 sample AND - qPCR negative on D1 sample AND - culture negative on D1 sample | Meets inclusion criteria but not probable or confirmed |

2. **Interventions: Dose and Duration**

During the initial enrolment period when suspected cases were randomised:

Ciprofloxacin:

- Adults: 500mg orally twice daily (or 400mg IV twice daily for those who cannot take oral medication) for 10 days.
- Children: 15mg/kg twice daily (max 500mg per dose) orally (or 10mg/kg IV twice daily for those who cannot take oral - maximum dose 400mg) for 10 days.
- Patients who begin intravenous therapy may switch to oral administration once they are able to swallow or once deemed clinically appropriate by the treating physician.

Streptomycin + ciprofloxacin:

- Adults: Streptomycin 1 g twice daily for 5 days with ciprofloxacin 500 mg orally twice daily (or ciprofloxacin 400 mg twice daily IV for those who cannot take it orally) for 10 days.
- Children: streptomycin 15mg/kg twice daily for 5 days with ciprofloxacin 15mg/kg twice daily (max 500mg per dose) orally (or 10mg/kg IV twice daily for those who cannot take it orally - maximum dose 400mg) for 10 days. This is the alternative treatment proposed by the Ministry of Public Health since the 2018-2019 plague season.
- Patients who start taking intravenous ciprofloxacin may switch to oral administration once they are able to swallow or once deemed clinically appropriate by the treating physician.

Gentamicin + ciprofloxacin:

- Adults: gentamicin 2.5mg/kg IV twice daily for 5 days with ciprofloxacin 500 mg orally twice daily (or ciprofloxacin 400 mg twice daily IV for those who cannot take it orally) for 10 days.
- Children: gentamicin IV 2.5mg/kg twice daily for 5 days with ciprofloxacin 15mg/kg twice daily (max 500mg per dose) orally (or 10mg/kg IV twice daily for those who cannot take it orally - maximum dose 400mg) for 10 days.

During the subsequent observation cohort period:

Gentamicin + ciprofloxacin as above (Streptomycin + ciprofloxacin was also permissible under the protocol, although global production of Streptomycin had ceased)

3. **Participant Flow**

Supplementary Figure 1. Participants' flow
